# Pregnancy with multiple high-risk factors: a systematic review and meta-analysis

**DOI:** 10.7189/jogh.15.04027

**Published:** 2025-02-07

**Authors:** Yue Zhang, Weijie Ding, Tingting Wu, Songtao Wu, Hui Wang, Muhammad Fawad, Akilew Awoke Adane, Xiaochen Dai, Xiaoqin Zhu, Xiaolin Xu

**Affiliations:** 1 School of Public Health, The Second Affiliated Hospital, Zhejiang University School of Medicine, Hangzhou, Zhejiang, China; 2 Health Care Department, Huai’an Maternal and Child Health Care Hospital Affiliated to Yangzhou University, Huai’an, Jiangsu, China; 3 School of Medicine, Zhejiang University, Hangzhou, Zhejiang, China; 4 Ngangk Yira Institute for Change, Murdoch University, Murdoch, Australia; 5 Telethon Kids Institute, The University of Western Australia, Nedlands, Australia; 6 Department of Health Metrics Science, School of Medicine, University of Washington, Seattle, USA; 7 Institute for Health Metrics and Evaluation, University of Washington, Seattle, USA; 8 School of Public Health, Faculty of Medicine, The University of Queensland, Brisbane, Australia

## Abstract

**Background:**

A wide spectrum of high-risk factors in pregnancy can lead to adverse pregnancy outcomes or short- or long-term health effects. Despite this, there has been no synthesis of findings on the measurement, potential causes, and health outcomes of multiple high-risk factors in pregnancy (MHFP). We aimed to address this gap by summarising the existing research on this topic.

**Methods:**

We retrieved studies published up to 3 June 2024 through systematic database searches and used a narrative synthesis approach to summarise the measurement, patterns, causes, and outcomes of MHFP. We also estimated the pooled MHFP prevalence through meta-analysis with a random effects model and performed subgroup analyses and meta-regression to examine potential sources of between-study heterogeneity.

**Results:**

We included 83 observational studies published between 2010 and 2024, of which 72% were from high-income countries. These studied factors can be grouped into four categories: physical conditions, mental conditions, sociobehavioural problems, and pregnancy history. We identified 16 MHFP patterns, among which co-existing multiple physical conditions were the most common pattern. The overall pooled prevalence of MHFP was 12% (95% confidence interval (CI) = 12–13), with an increasing trend and relatively higher levels in low- and middle-income countries (LMICs). We observed heterogeneity in the measurement of MHFP across the studies, possibly due to the number of risk factors in the definition of MHFP. About 78% of included studies investigated MHFP-associated health outcomes for women and offspring, with only two studies examining long-term maternal or offspring outcomes later in life.

**Conclusions:**

Research into MHFP has been emerging over the past decade, but is far from complete. The burden of MHFP is increasing worldwide, particularly LMICs. Maternal healthcare systems must shift to a multidisciplinary and integrated framework so as to better design and implement prevention and intervention programmes and sustain the healthy development of the next generation.

**Registration:**

PROSPERO: CRD42022358889.

Global fertility rates have been declining for decades, leading to shifts in demographic patterns worldwide [1]. In this context, the maternal mortality rate is rising again in many countries [2,3]. Around 810 women died from preventable causes related to pregnancy and childbirth every day in 2020, with 94% of these deaths occurring in low- and middle-income countries (LMICs) [4]. A wide spectrum of high-risk factors in pregnancy has been identified to affect maternal health [5–8], including pre-existing health conditions, pregnancy-related health conditions and complications, lifestyle factors, and younger or advanced maternal age [9]. Some of these were suggested to contribute to the higher rate of maternal pregnancy risk in recent years, such as the increased prevalence of advanced maternal age to the rise in chronic conditions [10]. These new challenges bring about concerns that the magnitude of high-risk factors in pregnancy is becoming increasingly diversified, emphasising the key need for maternal healthcare systems to dynamically meet the widening burden of pregnancy risk ensuing from demographic changes [11].

These transitions in demographic, socioeconomic, epidemiological, and environmental conditions lead to the possibility of the existence of multiple high-risk factors in pregnancy (MHFP) [11]. Women with MHFP may have difficulties in managing their complex health conditions and are at a higher risk of adverse pregnancy outcomes than those with single risk factors or healthy pregnancies [12–14]. Although the health effects of specific high-risk factors have been widely investigated individual, studies on MHFP are still limited. For example, a systematic review on maternal multimorbidity during pregnancy and after childbirth in LMICs found significant associations between different types of morbidities during pregnancy [15]. Another systematic review and meta-analysis showed that maternal multimorbidity was associated with multiple adverse birth outcomes [16]. These studies, however, only included a few common physical and mental conditions, failing to consider other risk factors, such as pregnancy history and sociobehavioural problems. There has also been no review or synthesis of evidence on MHFP thus far. This leaves a gap in our knowledge on a whole spectrum of high-risk factors in pregnancy and the patterns of their co-occurrence, as well as MHFP-related causes and health outcomes and possible interventions or management strategies. We therefore aimed to summarise the current evidence on the measurement, prevalence, causes, and health outcomes of MHFP.

## METHODS

### Search strategy and eligibility criteria

We searched PubMed, Embase, and Web of Science for articles published on the topic of MHFP from inception to 3 June 2024 using pre-designed search strings (Table S1 in the **Online Supplementary Document**). We followed the PRISMA 2020 statement in reporting our findings [17]. We included observational research (such as cross-sectional studies, case-control studies, nested case-control studies, and cohort studies) that measured at least two factors associated with maternal health; measured the co-existence of multiple factors simultaneously, rather each single factor separately; covered the definition, measurement, epidemiology, related causes, and outcomes of MHFP; enrolled pregnant women and/or their children; and written in English. We excluded studies that looked at risk factors initiated during the postpartum period, assessed the association between different types of high-risk factors rather than their co-existence, or had no full text available.

### Data extraction

Three authors (ZY, WT, WS) screened the titles/abstracts and full texts independently to select eligible articles, recording any exclusions reasons in the process. They met regularly to discuss the discrepancies with another researcher (XX). Following this stage, they extracted the following information from each study: title, author, publication year, design, country, research duration, sample size, number of participants with MHFP, objective, data collection tool, main exposure and outcome, measurements of MHFP, and main results related to MHFP (Table S2 in the **Online Supplementary Document**).

### Quality assessment

Three authors (ZY, WT, WS) independently assessed the methodological quality of the included studies using a quality assessment tool for observational studies developed by National Heart, Lung, and Blood Institute [18]. Any discrepancies were discussed with a third researcher (XX).

### Evidence synthesis

We used a narrative synthesis approach to describe the measurements and categories of high-risk factors in pregnancy and their co-existing patterns; the causes of MHFP; and the health outcomes associated with MHFP. Moreover, we also extracted the prevalence of MHFP from each study or calculated it as the proportion of pregnant women with multiple high-risk factors among all included pregnant women at the baseline. When prevalence was presented separately using subgroups within a single study, we first pooled the results using a meta-analysis method with fixed-effect models [19]. Then, we performed a meta-analysis to pool the prevalence of MHFP using a random effects model, considering that Cochran’s Q and *I*^2^ statistics suggested the existence of between-study heterogeneity. We then conducted subgroups analyses and meta-regression to investigate potential sources of between-study heterogeneity. Specifically, we included publication year, country origins of participants, country types by income, and sample sizes in the meta-regression analyses as independent variables, and we stratified our subgroup meta-analyses by the number of high-risk factors included in the MHFP definition, publication years, country origins of the participants, the income level of countries, and sample sizes. The trends of prevalence of MHFP stratified by income level of countries were estimated using linear regression.

We conducted the meta-analyses using Stata, version 16.0 (StataCorp LLC, College Station, TX, USA), and the visualisation using *R*, version 4.1.2 (R Core Team, Vienna, Austria).

## RESULTS

### Overview of search results

We retrieved 20 539 records from the databases, with 13 157 remaining after deduplication. After title and abstract screening, 362 studies remained for full-text review, with 83 included for analyses [12,13,20–100] (**Table 1****;** Figure S1 and Table S2 in the **Online Supplementary Document**). All of the included studies were published between 2010 and 2024, with most (n = 73) being published after 2016. About three-quarters (72%) of the studies were from high-income countries (HICs) [102], including the USA (n = 31), Canada (n = 8), the UK (n = 5), Germany (n = 3), Israel (n = 3), Portugal (n = 2), Finland (n = 2), Australia (n = 1), New Zealand (n = 1), Denmark (n = 1), Netherland (n = 1), Japan (n = 1), South Korea (n = 2), and France (n = 1). All included studies have declared how they measured MHFP; 54 (65%) reported the prevalence of MHFP, 66 (80%) discussed MHFP-related outcomes, and only 9 (11%) focussed on the potential causes of MHFP. Their sample sizes varied considerably, ranging from 47 to 73 109 790 women. Most studies were of moderate (n = 66) or high quality (n = 15).

**Table 1:** Basic characteristics of included articles

Article, year	Country	Participants and sample size	Study design	Measurements of multiple high-risk factors of pregnancy, their type, and their number	Quality
Tomlinson et al. [36], 2013	South Africa	1145 pregnant women	Cross-sectional study	Co-existing number of risk factors in pregnancy, including depression symptoms during pregnancy, HIV positive, alcohol use prior to pregnancy, and low birth weight of previous children (four risk factors)	Moderate
Yanit et al. [40], 2012	USA	532 088 women with singleton, non-anomalous deliveries	Retrospective cohort study	Co-existence of chronic hypertension and pre-gestational diabetes (two risk factors)	Moderate
Winkel et al. [39], 2015	Germany	283 pregnant women from gynaecological outpatient settings	Prospective longitudinal study	Comorbid anxiety disorders and depressive disorders prior to pregnancy (two risk factors)	Moderate
Wiencrot et al. [38], 2010	USA	176 845 pregnant women	Retrospective cohort study	Co-existence of mental illness/intentional injury or substance abuse/intentional injury or mental illness/intentional injury/substance abuse (three risk factors)	Moderate
Wainstock et al. [37], 2021	Israel	39 780 women with two first singleton consecutive deliveries	Retrospective nested case-control study	Number of complications during the first pregnancy, including small for gestational age, perinatal mortality, and preeclampsia (three risk factors)	Moderate
Somerville et al. [35], 2019	USA	1 185 182 delivery hospitalisations	Longitudinal study	Obstetric comorbidity index previously published by Bateman et al., which assigns a weight of 1–5 to each of 20 conditions identified from ICD-9-CM codes at delivery and maternal age ≥35 years old (20 risk factors)	Moderate
Shuffrey et al. [34], 2022	South Africa	600 maternal-infant dyads	Prospective study	Co-existence of prenatal depression and anxiety (two risk factors)	Moderate
Salahuddin et al. [33], 2019	USA	1 434 441 pregnant women	Cross-sectional study	Maternal comorbidity index developed by Bateman et al. derived the original index using comorbidities. The comorbidities that were included in the index were previous caesarean birth, gestational hypertension, mild or unspecified preeclampsia, pre-existing hypertension, asthma, severe preeclampsia, multiple gestation, pre-existing diabetes mellitus, drug abuse, placenta previa, chronic renal disease, HIV, cardiac valvar disease, systemic lupus erythematosus, sickle cell disease without crisis, congenital heart disease, alcohol abuse, pulmonary hypertension, chronic ischemic heart disease, and chronic congestive heart failure (20 risk factors)	Moderate
Reno et al. [32], 2021	USA	47 pregnant women	Retrospective cohort study	Co-occurring depression symptoms and high exposure to adverse social determinants (two risk factors)	Moderate
Premji et al. [31], 2020	Pakistan	300 pregnant women	Prospective cohort study	Co-existence of anxiety and depression (two risk factors)	Moderate
Obrochta et al. [30], 2020	USA and Canada	720 pregnant women	Prospective cohort study	Number of prenatal psychological distress, including depression, stress, and anxiety (three risk factors)	Moderate
Nunes et al. [29], 2020	Portugal	301 pregnant women	Retrospective cohort study	Co-existence of advanced maternal age with gestational diabetes, preeclampsia with gestational diabetes and maternal obesity with gestational diabetes (four risk factors)	Moderate
Ngocho et al. [28], 2019	Tanzania	200 pregnant women living with HIV	Cross-sectional study	Co-existence of depression, anxiety and HIV positive (three risk factors)	Moderate
McPherson et al. [27], 2012	USA	2106 twin pregnancies	Retrospective cohort study	Co-existence of twin pregnancies and early vaginal bleeding (two risk factors)	High
McDonald et al. [26], 2020	USA	1988 women who delivered during the study period	Retrospective cohort study	Number of psychosocial factors (three risk factors)	Moderate
Manoharan et al. [25], 2020	Australia	1545 women with gestational diabetes mellitus	Retrospective cohort study	Co-existence of polycystic ovary syndrome and gestational diabetes mellitus (two risk factors)	Moderate
Männistö et al. [24], 2016	USA	223 394 singleton pregnancies	Observational cohort study	Co-existence of depression, anxiety, and bipolar disease (three risk factors)	High
Maharlouei et al. [23], 2021	Iran	540 pregnant mothers	Cross-sectional study	Number of comorbidities, including hypothyroidism, diabetes mellitus/gestational diabetes mellitus, other endocrine disorders, headache, hypertension, cardiovascular diseases, immune thrombocytopenic purpura (seven risk factors)	Moderate
Ma et al. [22], 2022	China	2552 pregnant women in the third trimester	Prospective cohort study	Comorbid depression and anxiety. (2 risk factors)	Moderate
Lopez et al. [21], 2019	USA	178 737 pregnant women	Longitudinal study	Charlson comorbidity index, which provides a score for comorbidities for each discharged based on ICD-9-CM (17 risk factors in the index)	Moderate
Lee et al. [20], 2022	UK	71 522 pregnant women	Cross-sectional study	Presence of two or more pre-existing long-term physical or mental health conditions prior to pregnancy (79 long-term chronic conditions)	Moderate
Abdelwahab et al. [41], 2022	USA	507 pregnant women with opioid use disorder	Retrospective Cohort Study	Co-existence of opioid use disorder, psychiatric disorders, and hepatitis C infection (three risk factors)	High
Aoyama et al. [42], 2019	Canada	3 162 303 pregnant women	Nationwide population-based cohort study	Maternal comorbidity index (≥2) (34 chronic conditions)	Moderate
Asselmann et al. [43], 2018	Germany	533 pregnant women	Prospective-longitudinal study	Maternal anxiety and depressive disorders during pregnancy (two risk factors)	High
Berger et al. [44], 2021	USA	14 933 pregnant women	Retrospective study	The co-existence of advanced maternal age with hypertensive disorders/asthma/pre-gestational or gestational diabetes (five risk factors)	High
Bird et al. [45], 2017	New Zealand	6 822 pregnant women	Prospective cohort study	Two or more of the following risk factors: overweight or obese, weight gain during pregnancy, smoking status, doctor-diagnosed illness (diabetes, asthma, heart disease, high blood pressure, anaemia), disability, cigarette smoking, alcohol use, lack of regular activity (11 risk factors)	Moderate
Bond et al. [46], 2017	Canada	34 686 women with gestational diabetes during pregnancy	Retrospective cohort study	Co-existence of polycystic ovary syndrome and gestational diabetes mellitus (two risk factors)	High
Brown et al. [47], 2020	USA	1 480 925 women with deliveries	Retrospective cohort study	Co-existence of two or three or more comorbidities (29 diseases items)	High
Brown et al. [48], 2016	Canada	3932 pregnant women	Retrospective cohort study	Intellectual and developmental disabilities and mental Illness (two risk factors)	Moderate
Browne et al. [49], 2021	USA	3 097 123 pregnant women	Retrospective cohort study	Co-existence of pre-gestational diabetes with overweight, obesity or morbidly obese (two risk factors)	High
Chen et al. [50], 2021	Taiwan, China	10 614 pregnant women	Retrospective cohort study	Co-existence of perinatal depression and antidepressants. (two risk factors); co-existence of perinatal depression, antidepressants, anxiety disorder, multiple gestation, diabetes mellitus, hypertension, dyslipidaemia, reproductive tract infection, preeclampsia, placenta prevail, polycystic ovaries or pregnancy with history of infertility (12 risk factors)	Moderate
Choi et al. [51], 2022	South Korea	1 647 903 women	Retrospective cohort study	Co-existence of gestational diabetes with obesity, maternal age, family history of diabetes, hypertension, and insulin use (six risk factors)	Moderate
Christian et al. [52], 2021	USA	317 women (135 black, 182 white) in mid-pregnancy	Prospective study	Co-occurrence of depression and poor sleep quality during pregnancy (two risk factors)	Moderate
Cripe et al. [53], 2011	USA	3432 pregnant women	Prospective cohort study	Co-morbid mood-migraine disorders (two risk factors)	High
Cunningham et al. [54], 2017	USA	95 663 commercially insured women at low risk for caesarean delivery	Prospective cohort study	Co-existence of any two or more of the ten comorbidities (hypertension, including preeclampsia and eclampsia; diabetes; gestational diabetes; thyroid disorders; obesity; asthma; infections, such as HIV and toxoplasma; mental health conditions; substance use; and amniotic fluid issues including polyhydramnios and oligohydramnios) (10 comorbidities)	Moderate
Faulkner et al. [55], 2020	Canada	853 433 women with a singleton live birth and no recent mental healthcare	Observational cohort study	Co-existence of any two or more of the 16 chronic medical conditions (16 chronic conditions)	Moderate
Field et al. [56], 2010	Not report	911 pregnant women in second trimester of pregnancy	Prospective cohort study	Comorbid depression and anxiety (two risk factors)	Low
Flynn et al. [57], 2015	USA	419 pregnant women	Prospective cohort study	Co-existence of depression with anxiety, diabetes, high blood pressure or kidney problems; co-existence of anxiety with diabetes, high blood pressure or kidney problems (five risk factors)	Moderate
Friedman et al. [58], 2017	Peru	2922 pregnant women	Cross-sectional study	Co-existence of migraine and depression (two risk factors)	Moderate
Ghelichkhani et al. [59], 2021	Iran	98 pregnant women	Case-control study	Co-existence of COVID-19 and underlying diseases of heart conditions, diabetes, autoimmune diseases, chronic respiratory conditions, cancer, or asthma (seven risk factors)	Moderate
Hendryx et al. [60], 2014	USA	28 820 live singleton births	Cross-sectional study	Co-existence of not being married, stress during pregnancy, nulliparous, young mother, smoking, drug use, no prenatal care in first trimester, Medicaid coverage, being uninsured, less than high school education. (10 risk factors)	Moderate
Huang et al. [61], 2020	Taiwan, China	86 315 pregnant women	Retrospective cohort study	Preeclampsia comorbid with alcohol-related diseases, biliary stone, diabetes mellitus, hyperlipidaemia, hypertension, hepatitis B, hepatitis C, and the age of 36–45 years (nine risk factors)	Moderate
Hussamy et al. [62], 2019	USA	125 singleton pregnancies	Retrospective cohort study	Co-existence of the following risk factors: maternal age ≥35 years at delivery, abnormal aneuploidy screening, minor ultrasound marker(s), and sonographic identification of a major anomaly (four risk factors)	Moderate
Ibanez et al. [63], 2012	France	1719 women recruited before the 20th gestational week.	Observational cohort study	Comorbid depression and anxiety (two risk factors)	Moderate
Jardine et al. [64], 2020	England	276 766 women with a singleton birth	Cohort study	Age, BMI, pre-existing medical conditions, important obstetric history, and complications in current pregnancy (four risk factors)	Moderate
Kouhkan et al. [65], 2018	Iran	574 pregnant women	Prospective cohort study	Co-existence of assisted reproductive technology and gestational diabetes mellitus. (2 risk factors)	Moderate
Wang et al. [66], 2023	USA	3 757 582 pregnant women who gave live births	Retrospective cohort study	Co-existence of eclampsia and in vitro fertilisation (two risk factors)	High
Richardt et al. [67], 2023	Finland	375 pregnant women	Nested case-control study	Number of traditional and pregnancy-related risk factors including smoking, obesity, chronic hypertension, hypercholesterolemia, migraine, diabetes mellitus, gestational hypertension, eclampsia, and diabetes during pregnancy (nine risk factors)	Low
Kleinwechter et al. [68], 2022	Germany and Austria	1490 pregnant women with clinically confirmed COVID-19	Prospective observational study	Comorbid gestational diabetes, obesity and COVID-19 (three risk factors)	Moderate
Shuffrey et al. [69], 2022	USA and Puerto Rico	5822 pregnant women	Observational cohort study	Comorbid gestational diabetes and prenatal depression (two risk factors)	Moderate
Kuppusamy et al. [12], 2023	India	23 853 pregnant women	Cross-sectional survey	PMSMA guidelines: maternal risks: adolescent women whose ages ranged from 15 to 17 y; older women who were more than 35 y; women who were below 140 cm in height; women with a higher BMI. Lifestyle factors: smoking, using tobacco products besides cigarettes, and consuming alcohol. Medical risk: severely anaemic. Current health risks: had any comorbidities such as diabetes, hypertension, chronic respiratory diseases including asthma, thyroid disorders, heart diseases, cancer, and chronic kidney disorders; (5) Previous birth outcome risks: higher birth order (five and above), short birth spacing, long birth interval, a history of preterm deliveries, a history of adverse birth outcomes such as miscarriage, abortion, or stillbirth, most recent delivery was a caesarean section and were classified into the HRP group (21 risk factors)	Moderate
Mor et al. [70], 2024	Israel	1753 pregnant women	Retrospective cohort study	Coexisting gestational diabetes and preeclampsia (two risk factors)	High
Hou et al. [71], 2023	China	1082 participants receiving prenatal care	Longitudinal cohort study	Comorbid anxiety and depression (two risk factors)	Moderate
Du et al. [72], 2023	USA	72 183 pregnant women	Cross-sectional survey	Maternal comorbidity index (≥2) (21 risk factors)	Moderate
Yang et al. [73], 2023	Denmark	2 448 753 individuals	Longitudinal cohort study	Maternal hypertensive disorders and maternal diabetes history (two risk factors)	High
Azcoaga-Lorenzo et al. [74], 2023	UK	30 557 live births from 27 711 pregnant women	Retrospective cohort study	Multimorbidity was defined by the presence of two or more pre-existing long-term physical or mental health conditions. (79 risk factors)	Moderate
Jin Choi et al. [75], 2022	South Korea	5276 pregnant women	Case-control study	Coexisting maternal gestational diabetes and pre-pregnancy obesity (two risk factors)	Moderate
Brown et al. [76], 2023	Canada	2 014 508 pregnant women	Population-based cohort study	Twenty-two chronic conditions in pregnancy, including: asthma, cancer, cardiac arrhythmia, chronic hypertension, chronic liver disease, chronic obstructive pulmonary disease, congestive heart failure, coronary syndrome, diabetes mellitus, inflammatory bowel disease, mood and anxiety disorders, osteoarthritis, other mental illness, psychotic mental illness, renal failure, stroke, substance-use disorders, HIV, multiple sclerosis, obesity, rheumatoid arthritis and systemic lupus erythematosus (22 risk factors)	Moderate
Cao et al. [77], 2022	USA	950 315 pregnant patients in 2006 and 795 937 pregnant patients in 2016	Cross-sectional survey	Forty-one pre-gestational diagnoses or pregnancy-related complications, including: advanced maternal age, alcohol use, anaemia, asthma, chronic kidney disease, congenital heart disease, congestive heart failure, cystic fibrosis, diabetes mellitus, fibroid uterus, HIV, hypertension, ischemic heart disease, mental health, obesity, prior caesarean delivery, pulmonary hypertension, sickle cell disease/thalassemia+, substance abuse, systemic lupus erythematosus, tobacco use, valvular disease, chorioamnionitis, gestational diabetes, haemorrhage, infection, intrauterine fetal demise, intrauterine growth restriction, laceration (cervical, vaginal, perineal), large for gestational age, malpresentation, meconium, non-reassuring fetal heart tones, oligohydramnios, placental abruption, placental insufficiency, placenta previa, polyhydramnios, pregnancy-induced hypertension, pre-term delivery, and preterm premature rupture of membranes (41 risk factors)	Moderate
Pati et al. [78], 2022	India	127 pregnant women attending three antenatal clinics	Cross-sectional study	Multimorbidity was defined as the presence of two or more of 18 long-term conditions from which one is either: a physical non-communicable disease of long duration, like hypertension; a mental health condition of long duration, such as a mood disorder; an infectious disease of long duration, such as HIV (18 risk factors)	Moderate
Mateus et al. [79], 2022	Albania, Brazil, Bulgaria, Chile, Cyprus, Greece, Israel, Malta, Portugal, Spain, Turkey, and the UK	3326 pregnant women	Cross-sectional study	Comorbid depressive and anxiety symptoms (two risk factors)	Moderate
Maharjan et al. [80], 2024	USA	13 485 pregnant women who had a live birth	Nested case-control study	Maternal comorbidity index, severe preeclampsia, chronic congestive heart failure, sickle cell disease, multiple gestation, cardiac valvular disease, systemic lupus erythematosus, HIV, mild preeclampsia or unspecified preeclampsia, drug abuse, placenta previa, chronic renal disease, pre-existing hypertension, previous caesarean birth, gestational hypertension, alcohol abuse, asthma, pre-existing diabetes mellitus, and maternal age (19 risk factors)	Moderate
Bapayeva et al. [81], 2022	Kazakhstan	323 diabetic pregnant women	Cross-sectional study	Coexisting advanced maternal age and obesity in pregnant women with diabetes (three risk factors)	Moderate
Zhang et al. [82], 2023	China	2942 pregnant women who had live-birth singleton infants	1:1 matched case-control study	No folic acid supplementation during early pregnancy and preeclampsia (two risk factors)	Moderate
Kek et al. [83], 2023	Taiwan, China	2 297 613 pregnant women	1:4 matched cohort study	Gestational diabetes mellitus and hypertension (two risk factors)	Moderate
Zhou et al. [84], 2022	China	210 women with twin pregnancies	Cross-sectional survey	Co-morbid anxiety and depression (two risk factors)	Moderate
Wu et al. [85], 2024	China	149 women diagnosed with GDM (gestational diabetes mellitus)	Retrospective study	The co-existence of gestational diabetes mellitus and polycystic ovary syndrome (two risk factors)	Moderate
Hulsbosch et al. [86], 2023	Netherlands	1682 Dutch-speaking pregnant women with gestational age at birth ≥37 weeks, and without multiple pregnancy, severe psychiatric disorder or chronic disease history	Longitudinal prospective cohort study	Comorbid anxiety and depressive symptoms (two risk factors)	Moderate
Nakanishi et al. [87], 2023	Japan	86 674 singletons pregnant women	Cohort study	Maternal multimorbidity defined by: abnormal pre-pregnancy BMI, allergic disease, anaemia, diabetes mellitus, domestic violence, dyslipidaemia epilepsy, gastric or duodenal ulcer, heart disease, hepatitis, HIV infection, hypertension, inflammatory bowel disease, kidney disease, malignancy, migraine, neurological disease, other sexually transmitted diseases, psychiatric disorder, rheumatic or collagen disease, substance abuse, thyroid disease (23 risk factors)	Moderate
Brown et al. [88], 2023	USA	12 222 654 pregnant women	Cross-sectional survey	Maternal health conditions including anxiety-, bipolar-, depression-, disruptive/impulse control/conduct-, eating-, obsessive-compulsive disorder-, personality-, schizophrenia-, somatic-, suicidal ideation/attempt-, and traumatic/stress-related conditions (11 risk factors)	High
Meinhofer et al. [89], 2022	USA	20 914 591 pregnant women	Cross-sectional survey	Cannabis use disorder and substance use disorder (two risk factors)	Moderate
Echouffo Tcheugui et al. [90], 2022	Canada	886 295 pregnant women	Cohort study	Gestational diabetes and hypertensive disorders (two risk factors)	High
Wetcher et al. [91], 2023	USA	30 253 patients with nulliparous, term, singleton, vertex (NTSV) pregnancies	Cross-sectional survey	Points in OB-CMI score, including preeclampsia with severe features or eclampsia, preeclampsia/gestational/chronic hypertension, congestive heart failure, pulmonary hypertension, ischemic heart disease/cardiac arrhythmia, congenital heart and/or valvular disease, placental abruption, autoimmune disease/lupus, HIV/AIDS, sickle cell disease/bleeding disorder/coagulopathy/anticoagulation, epilepsy/cerebrovascular accident/neuromuscular disorder, chronic kidney disease, asthma, diabetes requiring insulin, maternal age, substance use disorder, alcohol abuse, and BMI (18 risk factors)	Moderate
Chen et al. [101], 2023	Finland	652 732 live births	Cohort study	Polycystic ovary syndrome (PCOS) and hypertensive disorders (two risk factors)	Moderate
Siqueira et al. [93], 2022	Brazil	15 105 hospitalised pregnant women with COVID-19	Cohort study	COVID-19, diabetes, obesity, cardiovascular disease, asthma, immunosuppression, renal disease, pulmonary disease, neurological disease, haematological disease, and liver disease (11 risk factors)	Moderate
Sweeney et al. [94], 2024	USA	40 840 deliveries	Retrospective cohort study	Chronic hypertension and hypertensive disorders (two risk factors)	Moderate
Shen et al. [95], 2024	China	1941 pregnant women over 18 years of age	Population-based longitudinal study design	Comorbid anxiety and depression (two risk factors)	Moderate
Mussa et al. [96], 2023	Canada	431 980 mothers with two consecutive, singleton deliveries	Retrospective cohort study	Gestational diabetes and hypertension (two risk factors)	Moderate
Mhereeg et al. [97], 2023	Wales	25 111 women eligible for vaccination during pregnancy	Cohort study	Depression, diabetes, asthma, and cardiovascular disease (four risk factors)	Moderate
Linder et al. [98], 2022	USA	73 109 790 delivery hospitalisations	Repeated cross-sectional analysis	Congenital heart disease, congestive heart failure, arrhythmia, valvular disease, pulmonary disorders, and thromboembolism (six risk factors)	Moderate
Akinsolu et al. [99], 2023	Ibadan, Nigeria	402 pregnant women of known HIV	Cross-sectional survey	Co-occurrence of depression and perceived stress (two risk factors)	Moderate
Li et al. [13], 2023	China	135 274 pregnant women	Multicentre retrospective cohort study	Pregnancy multimorbidity with eight types of pregnancy complications, including gestational diabetes mellitus, gestational hypertension, preeclampsia, postpartum haemorrhage, placental previa, placental abruption, infection, and severe anaemia (eight risk factors)	High
Stanhope et al. [100], 2022	USA	14 225 births	Retrospective cohort study	Psychiatric history, pre-existing cardiac disease, HIV, connective tissue or autoimmune disorder, bleeding disorder, chronic kidney disease, pulmonary hypertension, chronic diabetes (type I or II), chronic hypertension, seizure disorders, and asthma (11 risk factors)	Moderate

BMI – body mass index, HRP – high-risk pregnancy, ICD-9-CM – International Classification of Diseases, 9th Revision, Clinical Modification, OB-CMI – obstetric comorbidity index

### Definition, measurement, and patterns of MHFP

There has not been an international consensus on the definition of MHFP. Here we reviewed studies that focussed on the co-existence of two or more high-risk factors in pregnant women. The number of high-risk factors measured in the included studies varied, ranging from two single risk factors (*e.g.* gestational diabetes, hypertensive disorders) to a complex combination of risk factors (*e.g.* maternal multimorbidity including 79 chronic conditions). The occurring time of these factors during the perinatal period differed, as some factors were diseases existing before pregnancy, while others were complications occurring during pregnancy. Therefore, we classified these factors according to the time of the initiation of pregnancy [9,15] (**Table 2**): pregnancy history, high-risk factors existing before pregnancy, and high-risk factors occurring during pregnancy. The factors existing before or occurring during pregnancy were further classified into three subcategories according to their types: physical conditions, mental conditions, and sociobehavioural problems.

**Table 2:** Summary of measurements of multiple high-risk factors in pregnancy

Categories	High-risk factors in pregnancy
Pregnancy history	Low birth weight of previous infants; small for gestational age of the first pregnancy; preeclampsia of the first pregnancy; previous caesarean delivery; important obstetric history (including caesarean birth, uterine surgery, and others); pregnancy with history of infertility; order of index delivery ≥2nd; short birth spacing; long birth interval; a history of adverse birth outcomes such as Miscarriage, abortion, stillbirth, or perinatal mortality; most recent delivery was classified into the high-risk group
High-risk factors existing before pregnancy	
*Physical conditions before pregnancy*	Height <140 cm; HIV positive; chronic hypertension; Migraine; pre-gestational diabetes; polycystic ovarian syndrome; hypothyroidism; endocrine disorders; headache; cardiovascular diseases; congenital heart disease; immune thrombocytopenic purpura; inflammatory bowel disease; kidney problems; fibroid uterus; osteoarthritis; cancer; chronic respiratory diseases; chronic obstructive pulmonary disease; congestive heart failure; asthma; anaemia; chronic liver disease; biliary stone; overweight or obesity; dyslipidaemia/hyperlipidaemia/hypercholesterolemia; coronary syndrome; epilepsy; gastric or duodenal ulcer; systemic lupus erythematosus; stroke; connective tissue or autoimmune disorder; bleeding disorder; immunosuppression; rheumatoid arthritis; multiple sclerosis; thromboembolism; neurological disease; family history of diabetes mellitus; pre-existing multimorbidity or comorbidities (Charlson comorbidity index, number of chronic diseases, or number of comorbidities); hepatitis B/C infection; sexually transmitted diseases; sickle cell disease/thalassemia+
*Mental conditions before pregnancy*	Anxiety disorders prior to pregnancy; depressive disorders prior to pregnancy; mental illness prior to pregnancy; intellectual and developmental disabilities; psychiatric disorders
*Sociobehavioural problems before pregnancy*	Alcohol use prior to pregnancy; smoking prior to pregnancy
High-risk factors existing during pregnancy	
*Physical conditions or complications during pregnancy*	Advanced maternal age or maternal age ≤18; anaemia; twin pregnancy/multiple gestation; gestational diabetes; preeclampsia/eclampsia; gestational hypertension; early vaginal bleeding; excess weight gain/weight loss/no weight gain during pregnancy; nausea during pregnancy; disability during pregnancy; migraine disorders during pregnancy; COVID-19 infection during pregnancy; abnormal serum screening; abnormal ultrasonography (nuchal thickness 6 mm, echogenic intracardiac focus, echogenic bowel, renal pelvis dilatation, femur length < third percentile); detection of a major fetal anomaly; reproductive technology of this pregnancy; reproductive tract infection; preterm premature rupture of membranes; intrauterine fetal demise; intrauterine growth restriction; laceration (cervical, vaginal, perineal); malpresentation; meconium; non-reassuring fetal heart tones; oligohydramnios/polyhydramnios; placental abruption; placental insufficiency; placenta previa; chorioamnionitis; haemorrhage; complications during pregnancy or maternal multimorbidity (obstetric comorbidity index, or number of gestational complications)
*Mental conditions during pregnancy*	Depression symptoms during pregnancy; anxiety during pregnancy; mental illness during pregnancy; stress during pregnancy; bipolar disorder during pregnancy; mood disorders during pregnancy; poor sleep during pregnancy; disruptive/impulse control/conduct-related conditions; eating-related conditions; compulsive disorder-/related conditions; personality-related conditions; schizophrenia; suicidal ideation/attempt; traumatic/stress-related conditions
*Sociobehavioural problems during pregnancy*	Substance use disorders/abuse during pregnancy (*e.g.* drug abuse, alcohol abuse); smoking or tobacco use; cannabis use disorder; intentional injury during pregnancy; adverse employed status during pregnancy; poor income during pregnancy; food insecurity during pregnancy; housing instability during pregnancy; unacceptability of healthcare or prenatal care during pregnancy; unmarried; uninsured; low education level; unacceptability of social support during pregnancy; intimate partner violence/domestic violence during pregnancy; interpersonal trauma during pregnancy; lack of regular exercise during pregnancy; continuing to smoke during pregnancy; late prenatal care; use buprenorphine during pregnancy; using antidepressants during pregnancy; no folic acid supplementation during early pregnancy

**Figure 1:**
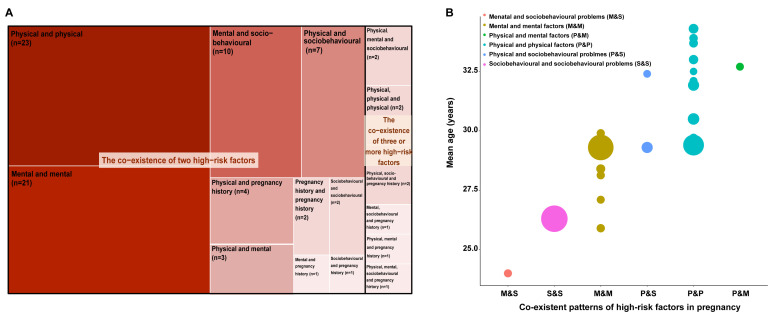
Patterns of co-existing multiple high-risk factors in pregnancy. **Panel A.** Tree map of the number of included studies for high-risk factors from different domains. **Panel B.** Mean gestational age of women according to included studies by different co-existing patterns of high-risk factors in pregnancy. Each circle refers to a study that mentioned the distribution of gestational age, from which the mean age of pregnant women with multiple factors was extracted. The size of each circle represents the number of pregnant women that had corresponding patterns of factors in the study.

We identified 16 types of MHFP patterns, among which co-existing physical conditions were the most common (23 studies), followed by co-existing mental conditions (21 studies), co-existing mental conditions and sociobehavioural problems (10 studies), co-existing physical conditions and sociobehavioural problems (seven studies), and co-existing physical and mental conditions (four studies). Nine studies measured co-existence of three or more factors (**Figure 1**, Panel A). Twenty-three studies reported the mean (standard deviation (SD)), median (quartile) or frequency distribution of maternal age among pregnant women with MHFP, allowing us to extract or calculate the mean age of pregnant women with specific MHFP patterns from each study. Pregnant women with co-existing physical and mental conditions (mean age = 32.7), existing physical and physical conditions (range of mean ages = 29.4–34.3 years, respectively), or existing physical and sociobehavioural problems (range of mean ages = 29.3–32.4 years) were generally older than those with co-existing mental conditions and sociobehavioural problems (mean age = 24.0 years) (**Figure 1**, Panel B). Specifically, the most common pair was the co-existence of anxiety and depression, which was reported by 14 studies, followed by advanced maternal age and gestational diabetes (n = 3), maternal obesity and gestational diabetes (n = 3), and chronic hypertension and pregestational diabetes (n = 3) (Table S3 in the **Online Supplementary Document**). The pairs of preeclampsia and gestational diabetes, as well as polycystic ovarian syndrome and gestational diabetes, were also reported by two studies.

### Prevalence of MHFP

**Figure 2:**
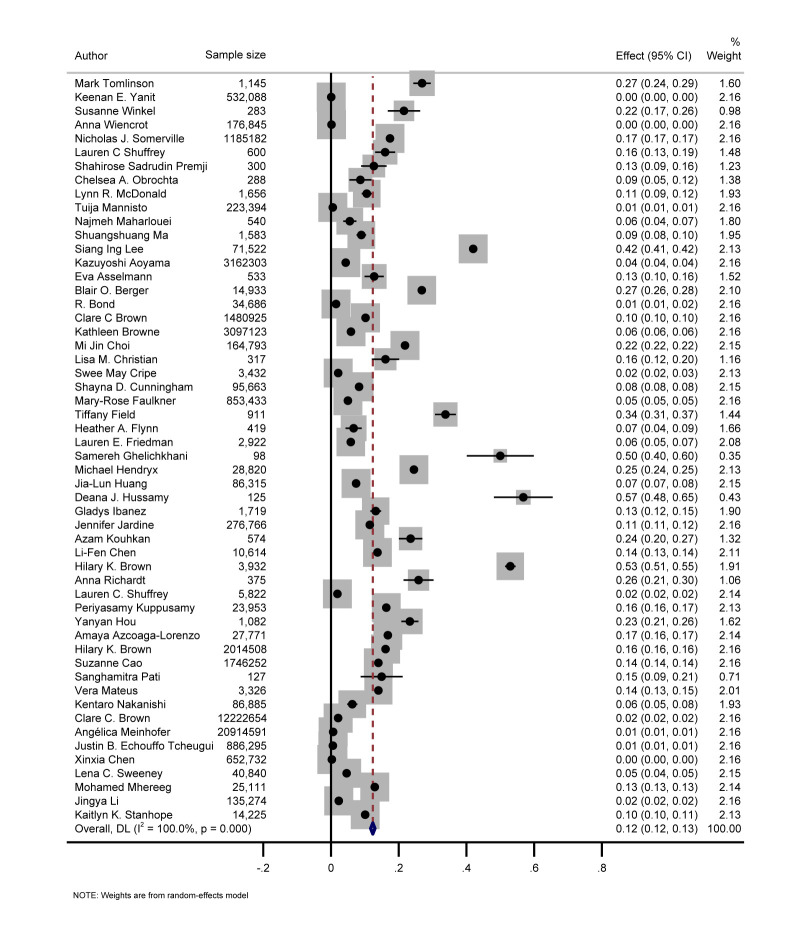
Random effects meta-analysis pooled prevalence estimates and forest plots for the co-existence of two or more risk factors in pregnancy.

**Figure 3:**
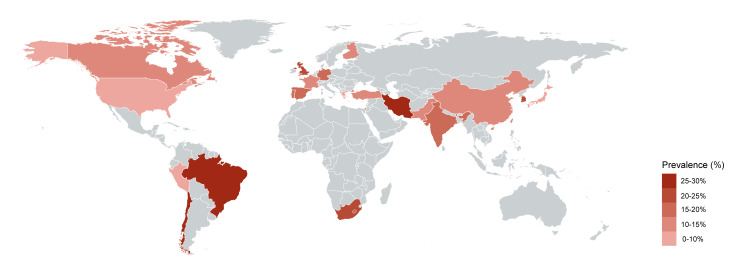
World map of the prevalence of co-existence of multiple high-risk factors in pregnancy.

The prevalence of MHFP among pregnant women was reported or could be calculated in 54 (65%) of studies. Their heterogeneity led to a wide range of prevalence from <1% to 57%. The pooled overall prevalence of MHFP was 12% (95% confidence interval (CI) = 12–13) (**Figure 2**). The meta-regression analysis revealed that a number of risk factors in the definition of MHFP could be a potential source of between-study heterogeneity (*P* = 0.021). We further conducted subgroup analyses stratified by the number of risk factors in MHFP (Figure S2 in the **Online Supplementary Document**) and found that the prevalence of MHFP was higher when the number of risk factors increased in the definition of MHFP (8% for two factors, 12% for three to 11 factors, and 15% for more than 12 factors). The prevalence of MHFP was similar in studies published before and after 2021 (Figure S3 in the **Online Supplementary Document**). We further conducted a subgroup analysis stratified by sample size (Figure S4 in the **Online Supplementary Document**) and found that the prevalence reported in studies with a larger sample size was relatively lower (7% for studies with ≥100 000 sample sizes and 21% for studies with ≤1000 sample sizes). We also extracted the prevalence of MHFP for each country from a single study or pooled it from several studies conducted in the same country by meta-analysis (**Figure 3**). The top four countries with the highest MHFP prevalence were Brazil (27%), Chile (26%), Iran (26%), and South Korea (21%), while the US (9%), Greece (9%), Cyprus (9%), Japan (6%), and Peru (6%) had a prevalence below 10%.

**Figure 4:**
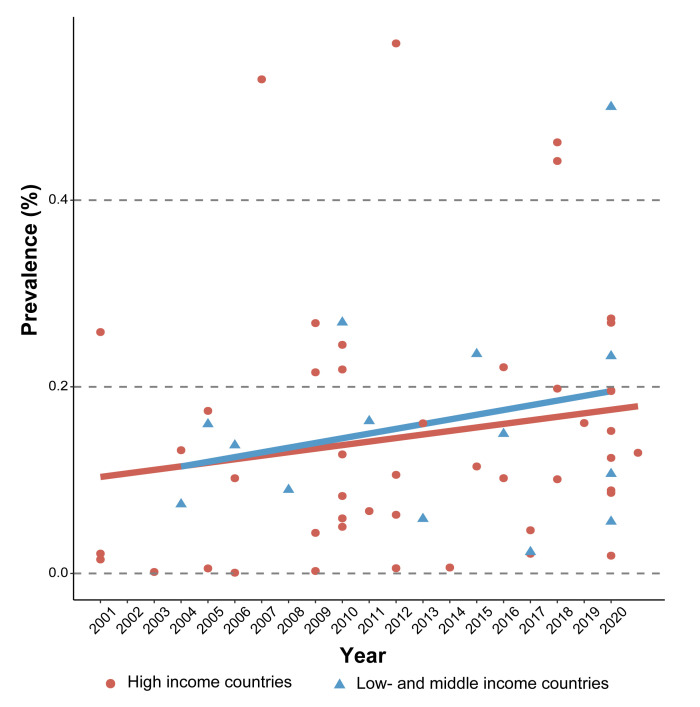
Global trend of the prevalence of co-existence of multiple high-risk factors in pregnancy according to included studies.

Thirty-eight of the 54 studies were from HICs, while 14 were from LMICs (one study did not report the country origin of the participants [56] and another included participants across nine countries [79]). The pooled prevalence of MHFP was relatively higher in LMICs (15%; 95% CI = 12–18) than in HICs (12%; 95% CI = 11–12) (Figure S5–6 in the **Online Supplementary Document**). Moreover, we found an overall increasing trend of MHFP from 2001 to 2020 in both HICs and LMICs (**Figure 4**). However, there was a mismatch between the high prevalence of MHFP and its publications, especially in LMICs, where the total number of publications was limited.

### Causes of MHFP

Only nine of the included studies focussed on the potential causes related to MHFP (**Table 3**). We grouped these causes into six categories: early-life experiences (adverse childhood experiences); adulthood adverse experiences (high level of perceived stress, stressful life events, lifetime experience of violence, being single or having dissatisfied marital relationship or partner status, lack of family support, and feeling HIV shame); socioeconomic factors (no education and lower income); lifestyle factors (smoking pre-conception and having raised body mass index); medical or physical factors (advanced maternal age, gestational age, and years on antiretroviral therapy); and pregnancy history (multigravidity and primiparity). These causes may overlap with several high-risk factors in pregnancy, such as smoking, having higher body mass index (BMI), and advanced maternal age, suggesting potential interaction effects across different high-risk factors.

**Table 3:** Summary of causes and health outcomes of the co-existence of multiple high-risk factors in pregnancy

Causes of the co-existence of multiple high-risk factors in pregnancy	Causes
Early-life experiences	Adverse childhood experiences
Adulthood adverse experiences	High level of perceived stress at any time point; stressful life events; lifetime experience of violence; being single/having dissatisfied marital relationship/partner status; lack of family support; HIV shame
Socioeconomic factors	No education; lower income
Lifestyle factors	Smoking pre-conception; having a higher body mass index
Medical or physical factors	Advanced maternal age; gestational age; years on antiretroviral therapy
Pregnancy history	Multigravidity; being primiparas
**Health outcomes of the co-existence of multiple high-risk factors in pregnancy**	**Causes**
Consequences for women	
*Health conditions during pregnancy*	Preeclampsia; hypertension during pregnancy; anxiety during pregnancy; severe maternal morbidity/cardiac severe maternal morbidity; caesarean; premature rupture of membranes; severe maternal course of COVID-19; maternal death due to COVID-19; having COVID-19 vaccination; maternal vascular malperfusion lesions; critical blood pressure during delivery hospitalisation; maternal ICU admission or death
*Postpartum health conditions*	Postpartum posttraumatic stress disorder; postpartum pancreatitis; postpartum haemorrhage/transfusion; postpartum psychological distress; ischemic stroke; postpartum depression; chronic inflammatory lesions; postpartum retinopathy; increased fasting glucose levels; chronic hypertension; postpartum diabetes/higher plasma glucose level; postpartum metabolic syndrome; hospital readmission; cardiovascular disease
Consequences for children	
*Fetal outcomes*	Poor fetal growth/intrauterine growth restriction; fetal Down syndrome; abruption; complicated birth; preterm birth; physiological birth; fetal vascular malperfusion lesions; thicker placentas; caesarean birth; spontaneous births
Neonatal health status	Intrauterine fetal demise/stillbirth; low score of 5-min APGAR; small for gestational age/low birth weight; large for gestational age/macrosomia; neonatal morbidity; neonatal intensive care unit admission; anatomical abnormalities
Infant health status	Toddlers’ sleep problem; infant interaction behaviours; childhood obesity; diabetes later in life

APGAR – appearance, pulse, grimace, activity and respiration, ICU – intensive care unit

### MHFP-associated health outcomes

Sixty-five studies investigated the MHFP-associated health outcomes (**Table 3**). These could be grouped into two categories: health outcomes for pregnant women and for children. For pregnant women, MHFP was associated with maternal conditions during pregnancy and postpartum health conditions. Again, the outcomes during pregnancy may overlap with high-risk factors in pregnancy (**Table 2**), suggesting the potential interaction effects among these health conditions. Regarding outcomes for children, MHFP could affect children’s health at multiple life stages, from the fetal to the neonatal and infant periods. Preterm birth was the most reported outcome of children associated with MHFP. However, due to the heterogeneity and differences in topics across included studies, we could not pool the effect sizes of MHFP on specific health outcomes through meta-analysis.

Specifically, most included studies focussed on short-term outcomes of pregnant women and their children, with only two studies examining long-term conditions, including increased risk of maternal cardiovascular disease [90] and offspring diabetes in later life [73]. A prospective cohort study with an overall follow-up period of 12.8 years found that, compared to women with no gestational diabetes and no gestational hypertensive disorder, those with co-occurrence of gestational diabetes and gestational hypertensive disorder were associated with a 2.4-fold higher risk of cardiovascular disease long-term after childbirth [90]. Another nationwide population-based study with a median follow-up of 19.3 years suggested that offspring of mothers with hypertensive disorders and comorbid diabetes during pregnancy were at 4.46-fold increased risk of developing diabetes, compared with those born to mothers with either only, or with neither hypertensive disorders nor diabetes history [73]. These studies extended the influence of MHFP beyond pregnancy and shortly after childbirth, providing further evidence of the associations of comorbid risk factors during pregnancy and long-term health risks for both women and offspring.

## DISCUSSION

To the best of our knowledge, this is the first review to explore the concept of MHFP and examine its global prevalence, potential causes, and associated health outcomes. According to our findings, the definition and types of high-risk factors in pregnancy are complex, multidimensional, and subjective. The pooled global prevalence of MHFP was 12% among the included studies, albeit with substantial heterogeneity that might have resulted from the number of risk factors in the definition of MHFP (*i.e.* the prevalence of MHFP was higher among studies with a higher number of risk factors). Subgroup analyses showed that the prevalence of MHFP was relatively higher in LMICs than in HICs; however, there was a mismatch between the high prevalence and research publications in LMICs. Heterogeneity also existed in MHFP-related topics across studies, limiting the meta-analysis for pooling the effect sizes of MHFP on specific health outcomes.

### Interpretations

We identified four dimensions of high-risk factors in pregnancy: physical conditions (both before and during pregnancy), mental conditions (both before and during pregnancy), sociobehavioural problems (both before and during pregnancy), and pregnancy history, which are similar to categories identified in prior studies [9]. However, there is an apparent lack of international standardisation and consensus for the definition of MHFP. Most included studies in our review focussed on the co-existence of two or three specific high-risk factors, failing to propose a definitive concept of MHFP. Also, high-risk factors measured in each of the included studies varied, making interpretation and comparison of findings across studies difficult. There is an urgent need for the establishment of an evidence-based approach that will define MHFP among a wide range of high-risk factors, as well as develop methods in data collection and analytical techniques to implement in the MHFP research.

Co-existing patterns of physical conditions were the most studied MHFP patterns according to our review, particularly for factors of gestational hypertension, preeclampsia/eclampsia, and gestational diabetes, suggesting that physical conditions remained the main research interest and the primary problem affecting maternal health [103]. More importantly, the increasing numbers of physical conditions within individuals may be multiplicatively associated with increased risks of adverse health outcomes. We further found the mean maternal age of women with co-existing physical conditions was relatively higher, implying the dual burden of physical conditions along with the delayed childbearing age [104–106]. Nevertheless, there are only a few studies on this topic, limiting the possibility for evidence-based implementation of clinical practice and policy making.

It is worth noting, however, that the number of studies focussing on the pattern of co-existing mental conditions and sociobehavioural problems is increasing, which were regarded as indirect factors affecting pregnancy outcomes [11,107]. These two types of factors frequently co-occur, but have been commonly neglected by current maternal healthcare services. For example, national guidelines for pregnant women, such as those in the USA [66], the UK [67], Australia [68], Canada [69], and Germany [12], have so far only taken mental disorders into a routine screening. Structured screening, effective interventions, and treatment options for mental disorders and sociobehavioural problems in pregnant women are still lacking. Yet, despite this increasing number of studies on the mental and sociobehavioural issues in pregnancy, we found that physical conditions still dominated research on MHFP. There is, therefore, a need for more investigations into these health problems and their interplays with physical conditions to provide further evidence for clinical practice. Moreover, the mean age of women with the co-existing patterns of mental conditions and sociobehavioural problems was relatively lower in our review, which is consistent with previous studies reporting the association of pregnancy at a young age with poor mental health outcomes [108]. Considering the variations of MHFP patterns across age groups, different strategies for maternal healthcare should be formulated targeting women in different age groups.

Our review pooled the prevalence of MHFP among included studies and found approximately 12% of pregnant women were influenced by MHFP worldwide, with this prevalence increasing over the past two decades. Some key factors would contribute to the increasing trend of MHFP, including the higher burden of certain chronic conditions [109], delayed maternal age [110], and the higher prevalence of unhealthy lifestyles among pregnant women [111]. However, compared with specific single risk factor (*e.g.* gestational diabetes and hypertension disorders), there is a mismatch between the high prevalence of MHFP and the underlying research. The first study was published in 2010 and most publications (64%) were only published after 2016, indicating the growing maternal health burden related to MHFP which initiated increasing research interest. According to the subgroups analysis, we found that the prevalence of MHFP increased with the higher number of factors included in the definition of MHFP, suggesting that our pooled prevalence of MHFP (12%) may be underestimated due to the heterogeneity in the measurement of MHFP. Future studies with a comprehensive measurement of MHFP are needed to obtain a more accurate estimate.

We found little variation in MHFP prevalence between country income groups. However, there was an imbalance in publications across countries, with significantly fewer studies from LMICs compared to HICs. Several points could have contributed to such mismatch, including the lack of clinical assessment tools or guidelines for identifying high-risk pregnancies [112], the lack of multidimensional clinical information [113], and limited funding in LMICs [114]. However, this finding could also be related to our exclusive review of English-language studies, which might have overlooked some LMICs and introduced geographic or income bias. The variation in number of publications also resulted in a huge variation of the estimated prevalence of MHFP in each single country. For example, we found a high prevalence of MHFP from studies conducted in Brazil, Chile, Iran, and South Korea (>25%), but a relatively low prevalence in the USA (<10%). Such prevalence was extracted or pooled from a few (*e.g.* 1–3) studies based on populations from Brazil, Chile, and Iran, which limited the generalisability of our findings and might have led to an inaccurate estimation of MHFP prevalence in these countries. Besides, the varied MHFP prevalence may also be explained by differences in the measured factors; diagnostic accuracy; the quality of antenatal, intranatal, and postnatal interventions; and socioeconomic differences across different regions.

For the types of maternal risk factors, infectious diseases (*e.g.* HIV/AIDS) remain one of the main focusses in LMICs such as Tanzania and South Africa [28,36]. The findings suggest that MHFP is especially debilitating in LMICs, where ending maternal deaths from single risk factors and infectious diseases is already a great challenge and remains the top priority in the maternal health policy agenda [4]. The unsolved problem of individual risk factors affecting maternal health may also hinder the progression of MHFP-related research in LMICs. Some sociobehavioural problems in pregnancy, such as poor income, food insecurity, and housing instability, were also identified in LMICs, indicating that women with low socioeconomic status are especially vulnerable to other subsequent factors. However, these factors may largely be neglected by current maternal healthcare work. Recognising and understanding inequalities, especially socioeconomic status, race, gender, and health insurance status, are crucial for ensuring all women’s rights to healthy pregnancy [4].

### Implications and recommendations

#### Moving from the focus on a single factor towards MHFP in future research

Compared with the single risk factor affecting maternal health (such as gestational diabetes, advanced maternal age, and hypertensive disorders during pregnancy), there is a mismatch between the high prevalence of MHFP and related research outputs. The findings of our review imply that many women are facing the convergency of multiple risk factors, rather than any single one. As suggested by some of the included studies, there is a need for future analysis to move from the focus on a single factor towards MHFP, exploring which high-risk factors often co-exist, what makes a woman at risk of developing MHFP, and how MHFP influences long-term health outcomes of women and their offsprings.

#### Developing a standardised definition and classification system for MHFP

Developing a standardised definition and classification system for MHFP will help ensure that research reports provide consistent data on MHFP, which will in turn facilitate the comparison and synthesis from multiple sources. Considering the current absence of a standardised methodology to define MHFP, it is important for future research to develop a uniform method for measuring MHFP from different perspectives, including public health surveillance, maternal and child healthcare systems, clinical practice, and perspectives of professionals and patients. The measurement should also consider the leading factors affecting maternal health in different social contexts when deciding which factors to be included in MHFP.

#### Conducting cohort studies and longitudinal analyses to understanding the causes and outcomes of MHFP, particularly long-term outcomes

We found only a few studies on the potential causes and outcomes related to MHFP, in particular for long-term outcomes after childbirth. The lack of cohorts with longitudinal follow-up of women from perinatal period to their later life and offsprings’ adulthood could be one of the reasons underlying this finding. It is important for researchers to make use of longitudinal data sets with long-term follow-up time to disentangle the causes and outcomes of MHFP, including the construction of predicting models of MHFP and the estimation of future health risks among those with MHFP [115]. Such results would be applicable in clinical practice for healthcare providers.

#### Catalysing multisectoral actions to provide integrated services for women with MHFP

Current clinical practice and national guidelines on maternal healthcare provide detailed suggestions for prevention, intervention, and treatment for some specific diseases or risk factors in pregnancy, such as HIV/AIDS, gestational diabetes, and gestational hypertension. However, there remain some neglected high-risk factors, including intentional injury, food insecurity, and housing instability, most of which are related to socioeconomic inequalities. Promoting access to healthcare services for all women, especially those in LMICs and those with low socioeconomic status, is important for ensuring all women’s rights to healthy pregnancy. Furthermore, collaborative maternal healthcare systems should catalyse multidisciplinary frameworks to provide integrated services to support women with MHFP, particularly in specific socioeconomic and cultural contexts [116], so as better design and implement prevention and intervention programmes to address MHFP and sustain the healthy development of next generation.

### Strengths and limitations

This is the first systematic review to synthesise evidence on co-existing high-risk factors in pregnancy and propose a working definition of MHFP. We used broad search strategies and inclusion criteria to identify more relevant studies. Several limitations, however, should also be pointed out in our methodology. First, despite the broad approach and our inclusion of a large number of articles, publication bias is still a possibility. Besides, there is no standard consensus on the definition and measurement of MHFP, therefore, we designed our search strategies based on the factors associated with maternal health, which might have led to us overlooking some studies. Second, we only included studies published in English, which could have led to linguistic, but also geographic and income bias, since the topic of maternal health is globally relevant, especially for LMICs, as they have a high prevalence of MHFP. Future research with more international collaborators could consider studies published in different languages, in particular for studies published in LMICs. Third, the heterogeneity among the included articles in terms of sample size, country of origin, and the measurement and definition of MHFP might have limited the reliability of pooled prevalence of MHFP. Besides, we could not conduct meta-analyses to quantify the association between MHFP and its related causes and health outcomes, particularly for long-term outcomes. Fourth, this study cannot address the potential confounding factors, such as socioeconomic status or access to healthcare, which would overlook the potential interactions between these factors and MHFP. This warrants further investigation.

## CONCLUSIONS

There is still a lack of international standardisation for the definition used to measure MHFP. While research into MHFP has been expanding over the past decade, it is still far from complete. Relevant studies, including systematic reviews, prospective cohort studies, and randomised control trials, are warranted to generate more evidence on MHFP to inform clinical practice and maternal health guidelines, particularly for LMICs. Maternal healthcare systems require a shift to a multidisciplinary and integrated framework to better design and implement prevention and intervention programmes targeting MHFP.

## Additional material


Online Supplementary Document
