# Supplementary material for: Pregnancy with multiple high-risk factors: a systematic review and meta-analysis
Source: J Glob Health. 2025 Feb 7;15:04027. doi: 10.7189/jogh.15.04027 (PMC11893144; doi:10.7189/jogh.15.04027)
Supplement: Online Supplementary Document [file jogh-15-04027-s001.zip › jogh-15-04027-s001.pdf]

Supplement to: Zhang Y, Ding W, Wu T, Wu S, Wang H, Faward M, Adane A, Dai X, Zhu X, Xu X. Pregnancy with multiple high-risk factors: a systematic review and meta-analysis. J Glob Health. 2025;15:04027.

Supplementary Tables 2
Table S1. Search strategies of the systematic review........ 2
Table S3. Number of studies for the co-existing patterns of high-risk factors in pregnancy........ 4
Supplementary Figures 7
Figure S1. Process of the study selection. 7
Figure S2. Subgroup meta-analysis pooled prevalence estimates and forest plots by the number of risk factors included in the definition of MHFP. 8
Figure S3. Subgroup meta-analysis pooled prevalence estimates and forest plots by published year. 9
Figure S4. Subgroup meta-analysis pooled prevalence estimates and forest plots by sample size........ 10
Figure S5. Subgroup meta-analysis pooled prevalence estimates and forest plots by income of participants’ countries........11
Figure S6. Subgroup meta-analysis pooled prevalence estimates and forest plots by participants’ countries. 12

Supplementary Tables

Table S1. Search strategies of the systematic review.

Database		Search Query	Results
PubMed	#1	(multiple risk factor*[Title/Abstract]) OR (multiple high risk factor*[Title/Abstract]) OR (multiple high-risk factor*[Title/Abstract]) OR (overlapping risk factor*[Title/Abstract]) OR (coexisting risk factor*[Title/Abstract]) OR (co-existing risk factor*[Title/Abstract]) OR (co-occurrence risk factor*[Title/Abstract]) OR (co-occur risk factor*[Title/Abstract]) OR (co-existence risk factor*[Title/Abstract]) OR (coexistence risk factor*[Title/Abstract]) OR (co-occurring risk factor*[Title/Abstract]) OR (cooccurring risk factor*[Title/Abstract])	15,477
	#2	(multiple complication*[Title/Abstract]) OR (overlapping complication*[Title/Abstract]) OR (coexisting complication*[Title/Abstract]) OR (co-existing complication*[Title/Abstract]) OR (co-occurrence complication*[Title/Abstract]) OR (co-occur complication*[Title/Abstract]) OR (accompanied complication*[Title/Abstract]) OR (multimorbid*[Title/Abstract]) OR (comorbid*[Title/Abstract]) OR (multiple condition*[Title/Abstract]) OR (multiple chronic condition*[Title/Abstract])OR (multiple morbid*[Title/Abstract]) OR (co-existence complication*[Title/Abstract]) OR (coexistence complication*[Title/Abstract]) OR (co-occurring complication*[Title/Abstract]) OR (cooccuring complication*[Title/Abstract])	315,770
	#3	syndemic[Title/Abstract]	1,364
	#4	#1 OR #2 OR #3	329,611
	#5	(pregnan*[Title/Abstract]) OR (maternal[Title/Abstract]) OR (maternity[Title/Abstract]) OR (gestation*[Title/Abstract]) OR (pre-pregnan*[Title/Abstract]) OR (perinatal[Title/Abstract]) OR (prenatal[Title/Abstract]) OR (obstetric[Title/Abstract]) OR (parturition[Title/Abstract])	1,049,962
	#6	#4 AND #5, Filters: humans, female, English.	5,964
Embase	#1	'multiple risk factor*':ti,ab,kw OR 'multiple high risk factor*':ti,ab,kw OR 'multiple high-risk factor*':ti,ab,kw OR 'overlapping risk factor*':ti,ab,kw OR 'coexisting risk factor*':ti,ab,kw OR 'co-existing risk factor*':ti,ab,kw OR 'co-occurrence risk factor*':ti,ab,kw OR 'co-occur risk factor*':ti,ab,kw OR 'co-existence risk factor*':ti,ab,kw OR 'coexistence risk factor*':ti,ab,kw OR 'co-occurring risk factor*':ti,ab,kw OR 'cooccurring risk factor*':ti,ab,kw	9,080
	#2	'multiple complication*':ti,ab,kw OR 'overlapping complication*':ti,ab,kw OR 'coexisting complication*':ti,ab,kw OR 'co-existing complication*':ti,ab,kw OR 'co-occurrence complication*':ti,ab,kw OR 'co-occurr complication*':ti,ab,kw OR 'accompanied complication*':ti,ab,kw OR multimorbid*:ti,ab,kw OR comorbid*:ti,ab,kw OR 'multiple condition*':ti,ab,kw OR 'multiple morbid*':ti,ab,kw OR 'co-existence complication*':ti,ab,kw OR 'coexistence complication*':ti,ab,kw OR 'co-occurring complication*':ti,ab,kw OR 'cooccurring complication*':ti,ab,kw	516,602
	#3	syndemic:ti,ab,kw	1,335
	#4	#1 OR #2 OR #3	526,134
	#5	pregnan*:ti,ab,kw OR maternal:ti,ab,kw OR maternity:ti,ab,kw OR gestation*:ti,ab,kw OR 'pre pregnan*':ti,ab,kw OR perinatal:ti,ab,kw OR prenatal:ti,ab,kw OR obstetric:ti,ab,kw OR parturition:ti,ab,kw	1,342,851
	#6	#4 AND #5 AND [female]/lim AND [humans]/lim AND [english]/lim	9,069
Web of Science	#1	AB=("multiple risk factor*" OR "multiple high risk factor*" OR "multiple high-risk factor*" OR "overlapping risk factor*" OR "coexisting risk factor*" OR "co-existing risk factor*" OR "co-occurrence risk factor*" OR "co-occur risk factor*" OR "co-existence risk factor*" OR "coexistence risk factor*" OR "co-occurring risk factor*" OR "cooccurring risk factor*") AND LA=(English)	4,765
	#2	TI=("multiple risk factor*" OR "multiple high risk factor*" OR "multiple high-risk factor*" OR "overlapping risk factor*" OR "coexisting risk factor*" OR "co-existing risk factor*" OR "co-occurrence risk factor*" OR "co-occur risk factor*" OR "co-existence risk factor*" OR "coexistence risk factor*" OR "co-occurring risk factor*" OR "cooccurring risk factor*") AND LA=(English)	472
	#3	#1 OR #2	5,033
	#4	AB=("multiple complication*" OR "overlapping complication*" OR "coexisting complication*" OR "co-existing complication*" OR "co-occurrence	205,936

		complication*" OR "co-occur complication*" OR "accompanied complication*" OR "multimorbid*" OR "comorbid*" OR "multiple condition*" OR "multiple chronic condition*" OR "multiple morbid*" OR "co-existence complication*" OR "coexistence complication*" OR "co-occurring complication*" OR "cooccuring complication*") AND LA=(English)	
	#5	TI=("multiple complication*" OR "overlapping complication*" OR "coexisting complication*" OR "co-existing complication*" OR "co-occurrence complication*" OR "co-occur complication*" OR "accompanied complication*" OR "multimorbid*" OR "comorbid*" OR "multiple condition*" OR "multiple chronic condition*" OR "multiple morbid*" OR "co-existence complication*" OR "coexistence complication*" OR "co-occurring complication*" OR "cooccuring complication*") AND LA=(English)	25,200
	#6	#4 OR #5	209,028
	#7	AB=("syndemic") AND LA=(English)	898
	#8	TI=("syndemic") AND LA=(English)	497
	#9	#7 OR #8	1,003
	#10	#3 OR #6 OR #9	214,683
	#11	AB=("pregnan*" OR "maternal" OR "maternity" OR "gestation*" OR "pre-pregnan*" OR "perinatal" OR "prenatal" OR "obstetric" OR "parturition") AND LA=(English)	648,499
	#12	TI=("pregnan*" OR "maternal" OR "maternity" OR "gestation*" OR "pre-pregnan*" OR "perinatal" OR "prenatal" OR "obstetric" OR "parturition") AND LA=(English)	343,015
	#13	#11 OR #12	738,353
	#14	#10 AND #13	5,506

Table S3. Number of studies for the co-existing patterns of high-risk factors in pregnancy.

Co-existent patterns of high-risk factors in pregnancy	Number of studies
Anxiety and depression	14
Advanced maternal age and gestational diabetes	3
Maternal obesity and gestational diabetes	3
Chronic hypertension and pregestational diabetes	3
Pre-eclampsia and gestational diabetes	2
Polycystic ovarian syndrome and gestational diabetes	2
HIV and depression	1
Alcohol use and depression	1
HIV and alcohol use	1
Had a previous low birth weight infant and depression	1
HIV and had a previous low birth weight infant	1
Alcohol use and had a previous low birth weight infant	1
Mental illness and intentional injury during pregnancy	1
Intentional injury and substance abuse during pregnancy	1
Prenatal stress and anxiety	1
Twin pregnancy and early vaginal bleeding	1
Opioid use disorder and psychiatric disorders	1
Opioid use disorder and hepatitis C infection	1
Opioid use disorder and marijuana	1
Opioid use disorder and cocaine	1
Opioid use disorder and supplement of buprenorphine	1
Advanced maternal age and hypertensive disorders	1
Advanced maternal age and asthma	1
Intellectual and developmental disabilities and mental Illness	1
Perinatal depression and antidepressants	1
Gestational diabetes and family history of diabetes mellitus	1
Gestational diabetes and order of index delivery ≥2nd	1
Gestational diabetes and history of diabetes	1
Poor sleep and depressive symptoms during pregnancy	1
Mood and migraine disorders during pregnancy	1
Chronic medical condition and poverty	1
Prenatal depression and diabetes	2
Prenatal depression and high blood pressure	1
Prenatal depression and kidney problems	1
Anxiety and diabetes	1
Anxiety and high blood pressure	1
Anxiety and kidney problems	1
Migraine and depression	1
SARS-Cov-2-infected and with underlying diseases	1
Preeclampsia and alcohol-related disease	1
Preeclampsia and biliary stone	1
Preeclampsia and hyperlipidemia	1
Preeclampsia and hypertension	1
Preeclampsia and hepatitis B	1

Preeclampsia and hepatitis C	1
Preeclampsia and advanced maternal age	1
Advanced maternal age and abnormal aneuploidy serum	1
Advanced maternal age and minor ultrasound marker(s)	1
Abnormal aneuploidy serum and minor ultrasound marker(s)	1
Abnormal aneuploidy serum and anomaly	1
Minor ultrasound marker(s) and anomaly	1
Assisted reproductive technology and gestational diabetes mellitus during pregnancy	1
HIV, alcohol use prior to pregnancy, and depression during pregnancy	1
HIV, had a previous low birth weight infant, and depression	1
Alcohol use prior to pregnancy, had a previous low birth weight infant, and depression during pregnancy	1
HIV, alcohol use prior to pregnancy, and had a previous low birth weight infant	1
Mental illness during pregnancy, intentional injury during pregnancy and substance abuse during pregnancy	1
Prenatal depression, stress and anxiety	1
Depression, anxiety and HIV	1
Bipolar disorder with depression and anxiety during pregnancy	1
Perinatal depression, antidepressants and anxiety disorder	1
Perinatal depression, antidepressants and multiple gestation	1
Perinatal depression, antidepressants and diabetes mellitus	1
Perinatal depression, antidepressants and hypertension	1
Perinatal depression, antidepressants and dyslipidemia	1
Perinatal depression, antidepressants and reproductive tract infection	1
Perinatal depression, antidepressants and pre-eclampsia	1
Perinatal depression, antidepressants and placenta previa	1
Perinatal depression, antidepressants and polycystic ovaries	1
Perinatal depression, antidepressants and pregnancy with history of infertility	1
Advanced maternal age, abnormal aneuploidy serum and minor ultrasound marker(s)	1
Advanced maternal age, abnormal aneuploidy serum and anomaly	1
Abnormal aneuploidy serum, minor ultrasound marker(s) and detection of a major fetal anomaly	1
Small for gestational age of the first pregnancy, perinatal mortality of the first pregnancy and preeclampsia of the first pregnancy	1
Obesity, advanced maternal age, and parity ≥4	1
HIV, alcohol use prior to pregnancy, had a previous low birth weight infant and depression during pregnancy	1
Depression symptoms and more than four adverse social determinants	1
High stress, smoking, drug use, unmarried, medicated, and late prenatal care	1
Previous caesarean section, obesity, pre-existing medical conditions, important obstetric history, important obstetric history	1
Unmarried, first birth, young mother (age ≤18), low education and medicated	1
Multimorbidity defined by 79 conditions	1
Maternal comorbidity index defined by 21 chronic conditions	1
Eclampsia and in vitro fertilization	1
Smoking, obesity, chronic hypertension, hypercholesterolemia, migraine, diabetes mellitus, Gestational hypertension, eclampsia, and diabetes	1
Comorbid gestational diabetes, obesity and COVID-19	1
21 risk factors in PMSMA guidelines	1
Gestational diabetes and preeclampsia	1
Maternal hypertensive disorders and maternal diabetes history	1
Coexisting maternal gestational diabetes and pre-pregnancy obesity	1
22 chronic conditions in pregnancy including: asthma, cancer，etc.	1
41 pre-gestational diagnoses or pregnancy-related complications	1
Multimorbidity defined as the presence of two or more of 18 long-term conditions	1
19 factors included in maternal comorbidity index	1
Coexisting advanced maternal age and obesity in pregnant women with diabetes	1
No folic acid supplementation during early pregnancy and preeclampsia	1
Co-morbid anxiety and depressive	1

Multimorbidity with eight types of pregnancy complications	1
The co-existence of gestational diabetes mellitus and Polycystic ovary syndrome	1
Maternal multimorbidity defined 23 risk factors	1
11 metal health conditions	1
Cannabis use disorder and substance use disorder	1
Gestational diabetes and hypertensive disorders	1
OB-CMI score defined by 18 chronic conditions	1
Polycystic ovary syndrome (PCOS) and hypertensive disorders	1
Comorbidity defined by 11 risk factors	1
Chronic hypertension and hypertensive disorders	1
Gestational diabetes and hypertension	1
Depression, diabetes, asthma, and cardiovascular disease	1
Congenital heart disease, congestive heart failure, arrhythmia, valvular disease, pulmonary disorders, and thromboembolism	1
Depression and perceived stress	1
Pregnancy multimorbidity with eight types of pregnancy complications, including gestational diabetes mellitus (GDM), gestational hypertension, preeclampsia, postpartum haemorrhage	1
(PPH), placental previa, placental abruption, infection, and severe anaemia.	1
SMM defined by 11 risk factors	1

Supplementary Figures

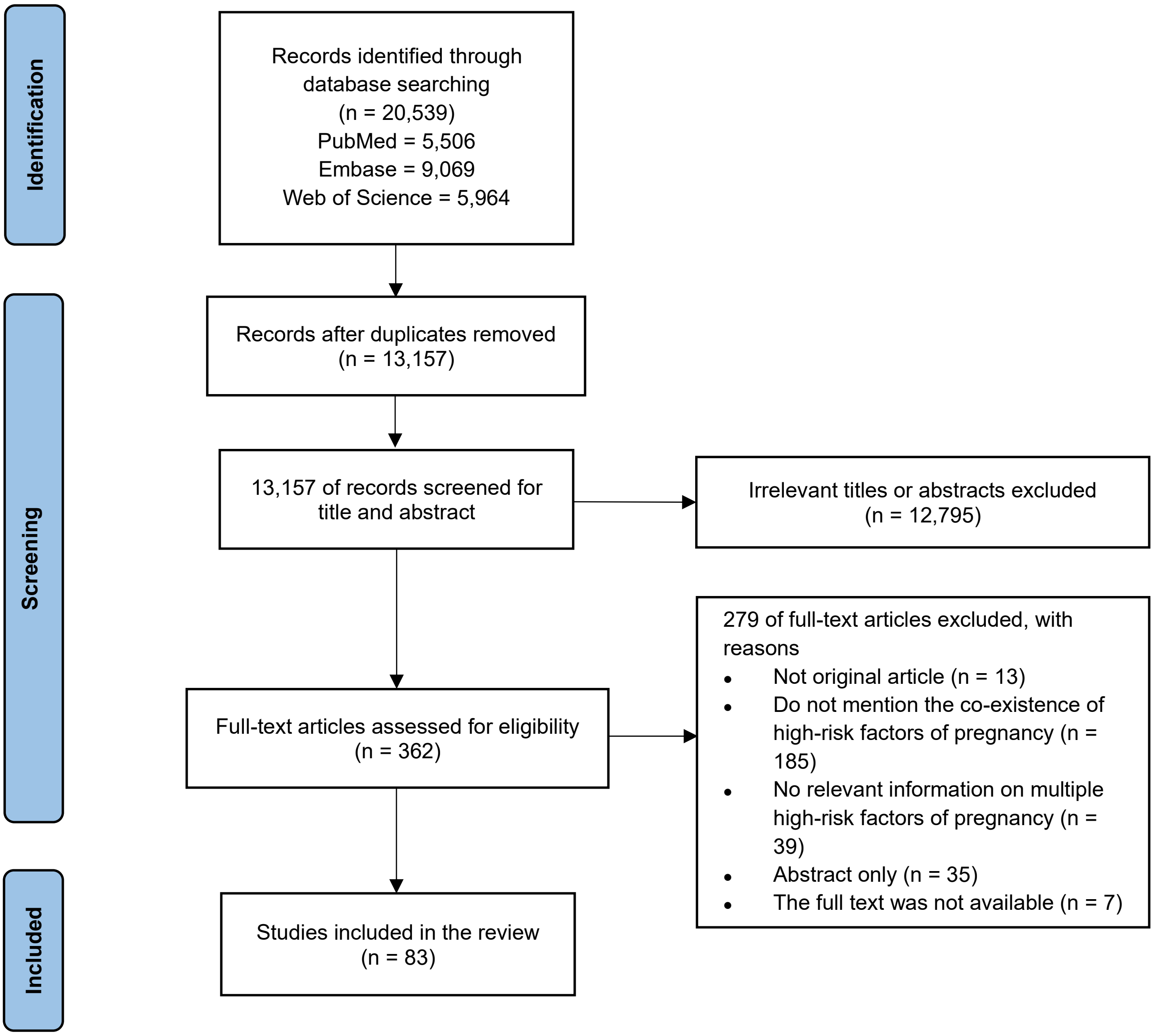

Figure S1. Process of the study selection.

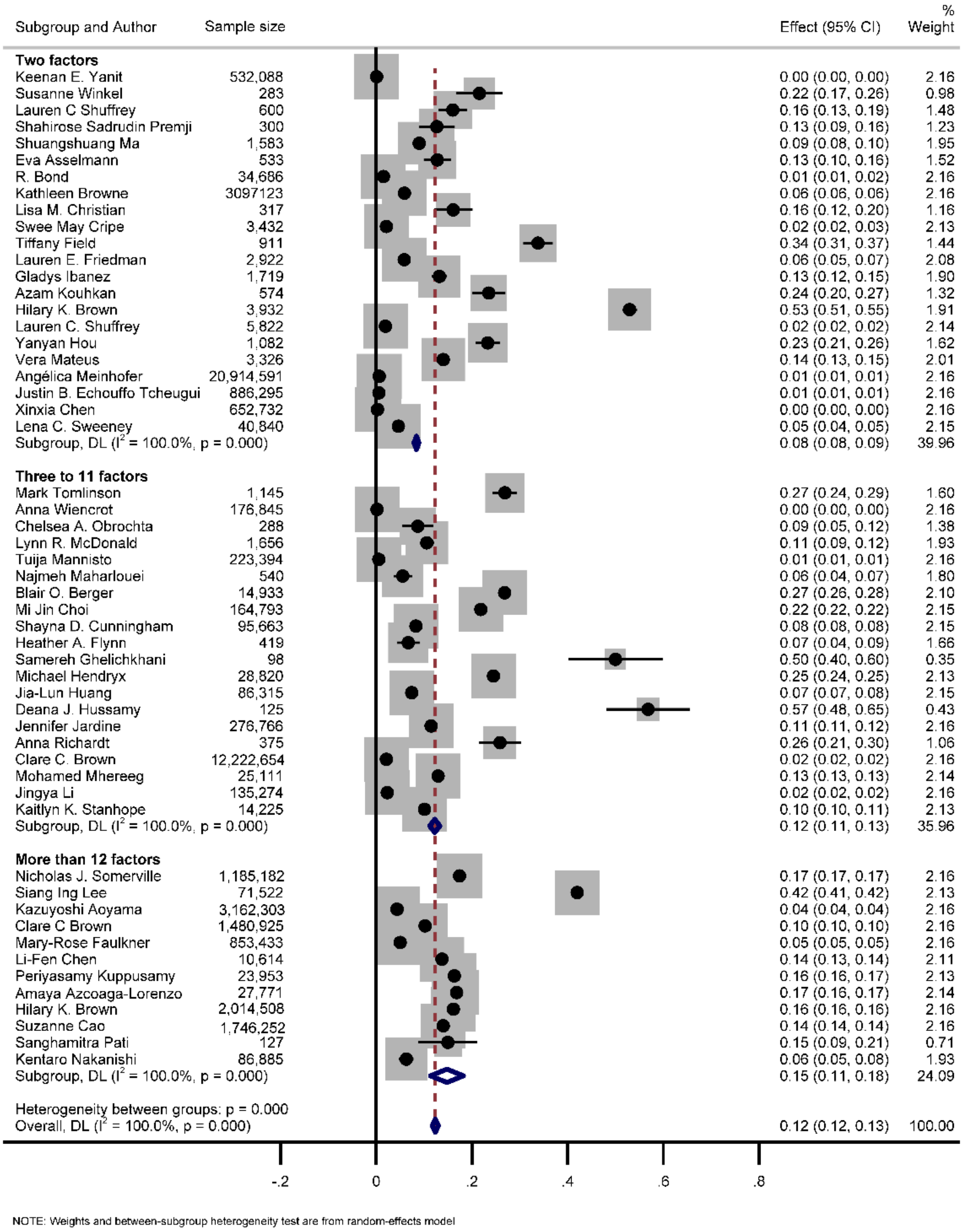

Figure S2. Subgroup meta-analysis pooled prevalence estimates and forest plots by the number of risk factors included in the definition of MHFP.

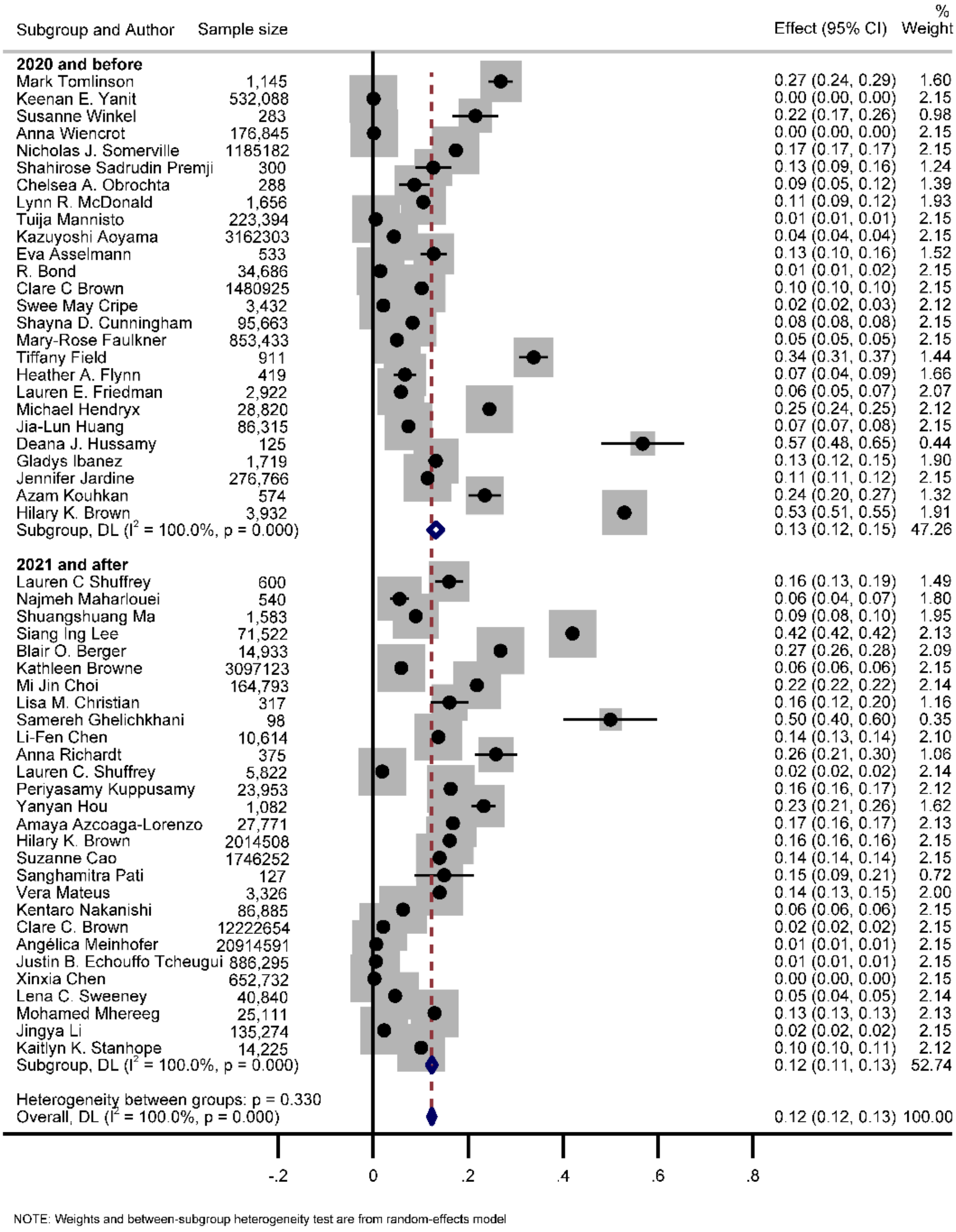

Figure S3. Subgroup meta-analysis pooled prevalence estimates and forest plots by published year.

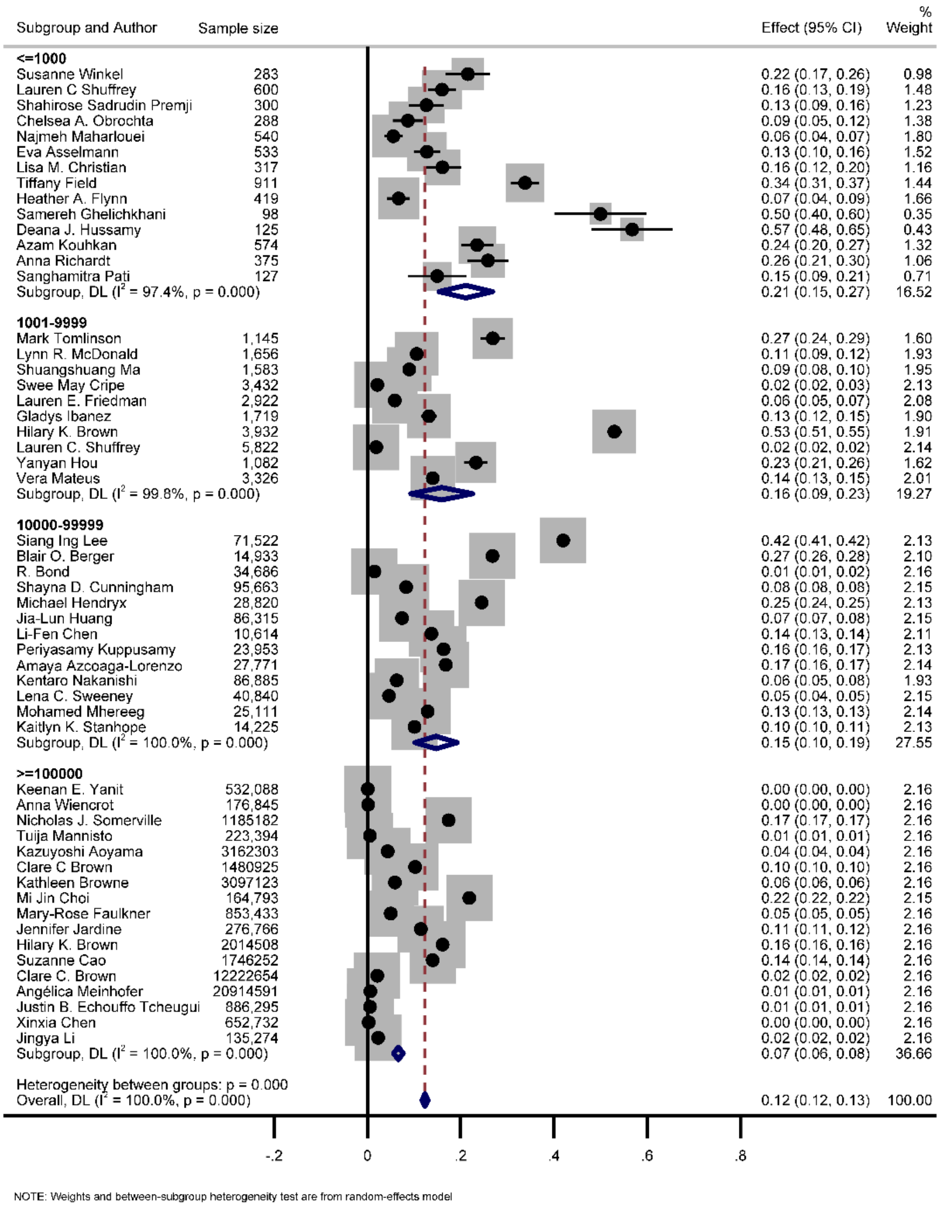

Figure S4. Subgroup meta-analysis pooled prevalence estimates and forest plots by sample size.

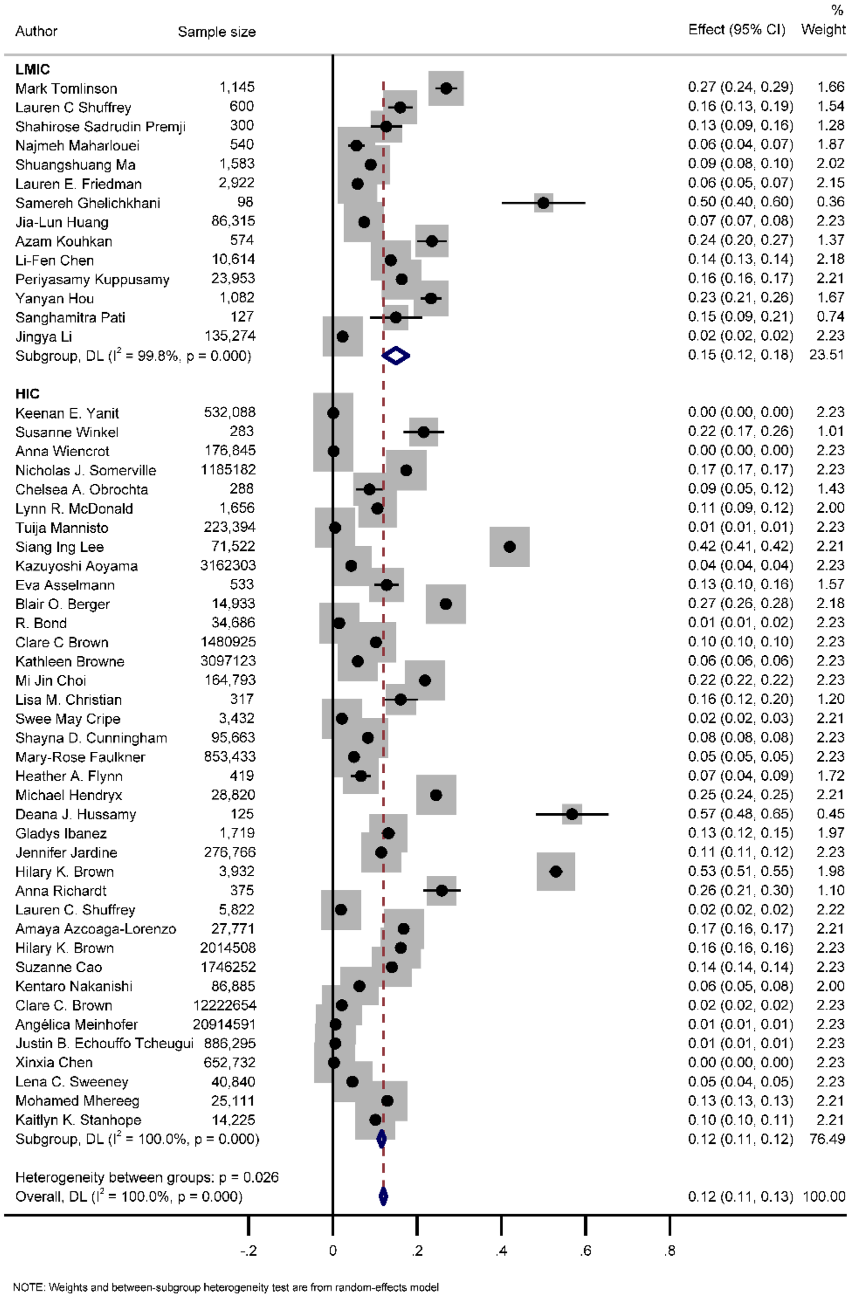

Figure S5. Subgroup meta-analysis pooled prevalence estimates and forest plots by income of participants' countries.

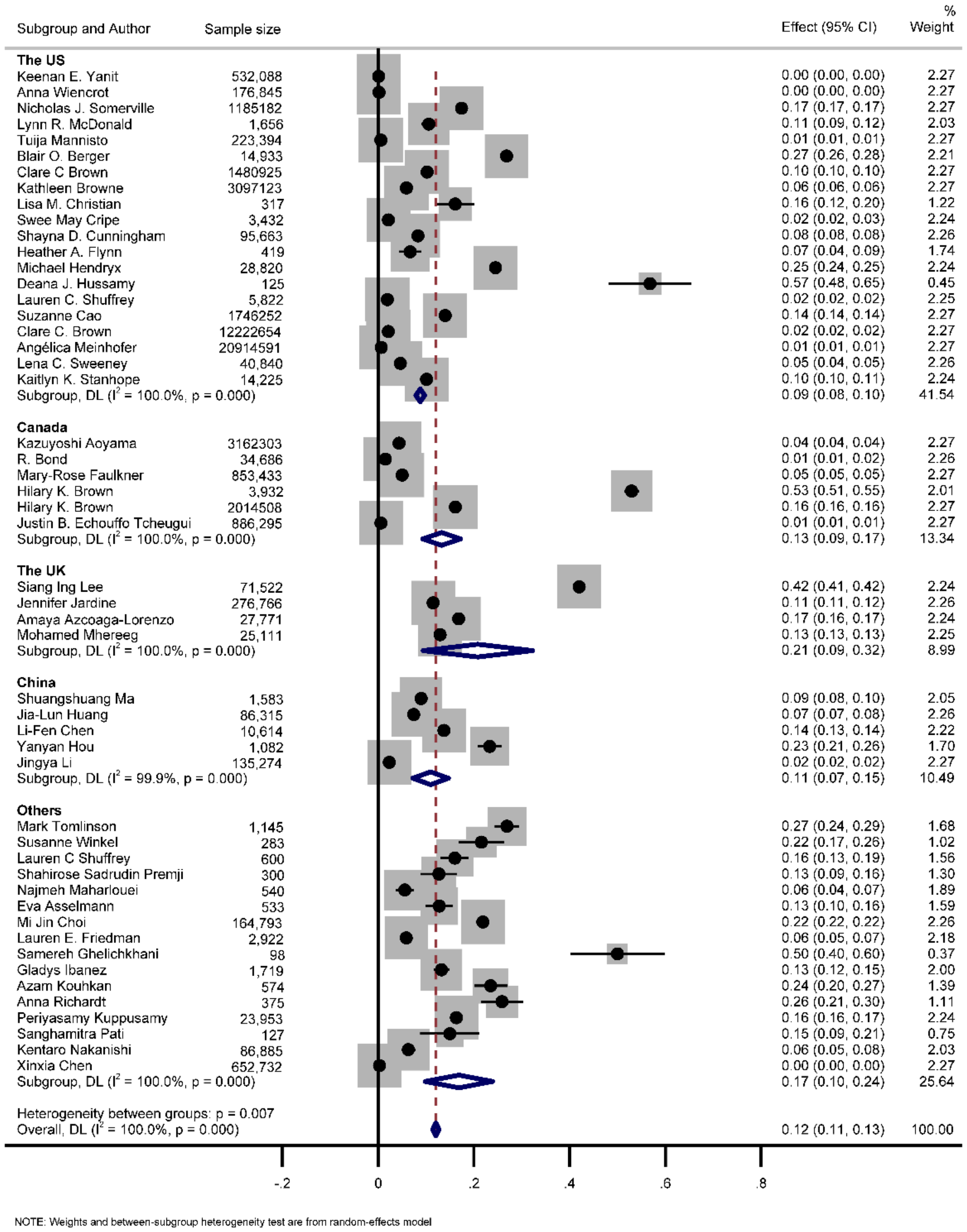

Figure S6. Subgroup meta-analysis pooled prevalence estimates and forest plots by participants' countries.
